# Silencing of the tRNA Modification Enzyme Cdkal1 Effects Functional Insulin Synthesis in NIT-1 Cells: tRNA^Lys3^ Lacking ms^2^- (ms^2^t^6^A_37_) is Unable to Establish Sufficient Anticodon:Codon Interactions to Decode the Wobble Codon AAG

**DOI:** 10.3389/fmolb.2020.584228

**Published:** 2021-02-09

**Authors:** Amithi Narendran, Sweta Vangaveti, Srivathsan V. Ranganathan, Emily Eruysal, Miranda Craft, Omar Alrifai, Fu Yee Chua, Kathryn Sarachan, Breann Litwa, Sheetal Ramachandran, Paul F. Agris

**Affiliations:** ^1^ The RNA Institute and Department of Biological Sciences, University of Albany, Albany, NY, United States; ^2^ Program in Bioinformatics and Integrative Biology, University of Massachusetts Medical School, Worcester, MA, United States; ^3^ Knight Cancer Institute, Oregon Health Sciences, School of Medicine, Portland, OR, United States; ^4^ Department of Medicine, Duke University School of Medicine, Durham, NC, United States

**Keywords:** Type 2 diabetes, CDKAL1, tRNA modification, Cdkal1 protein, insulin, Wobble decoding on the ribosome

## Abstract

Human Genome Wide Association Studies found a significant risk of Type 2 Diabetes Mellitus (T2DM) in single nucleotide polymorphisms in the *cdkal1* gene. The *cdkal1* gene is remote from the insulin gene and with the surprising function of a specific tRNA modification. Population studies and case control studies acquired evidences of the connection between Cdkal1 protein and insulin production over the years. To obtain biochemical proofs directly linking potential SNPs to their roles in insulin production and availability is challenging, but the development of Cdkal1 knock out mice and knock out cell lines made it possible to extend our knowledge towards therapeutic field of diabetic research. Supporting the evidences, here we show that knock down of the *cdkal1* gene using small interfering and short hairpin RNA in the NIT-1 cell line, a β-cell line inducible for insulin resulted in reduced levels of *cdkal1* and mature insulin mRNAs, increased the level of precursor insulin mRNA, decreased Cdkal1 and insulin proteins, and diminished modification of tRNA^Lys3^ from t^6^A_37_ to ms^2^t^6^A_37_, the specified function of Cdkal1. tRNA^Lys3^ lacking ms^2^- is incapable of establishing sufficient hydrogen bonding energy and hydrophobic stabilization to decode the wobble codon AAG.

## Introduction

Type 2 Diabetes Mellitus (T2DM) accounts for 90% of the 34 million diabetes cases in the US (Centers for Disease Control and Prevention, 2020). A fundamental genetic heterogeneity has been found in T2DM (Mannino et al., 2019). Genome Wide Association Studies (GWAS) focused on the genetic heterogeneity have discovered a number of loci, *KLF14, KCNQ1, DUSP9, FTO, HNF4A, IGFBP2, CDKN2A/B, TCF7L2, KCNJ11,* antioxidant genes, *DNAJC3, PGC-1α, ADIPOQ, CDKAL1, POMC, PPARγ2,* and *SLC30A8*, that could be potential predictors of the disease (Cho et al., 2019; Witka et al., 2019). One among the loci, in particular, CDK5 regulatory subunit associated protein 1-like 1, *cdkal1*, is intriguing as a locus remote from the insulin gene, and a tRNA modification enzyme. The gene product of *cdkal1*, Cdkal1 post-transcriptionally modifies tRNA to facilitate accurate translation of the insulin mRNA and processing of proinsulin to mature insulin protein (McCown, et al., 2020). Thus, Cdkal1 has been found to be a potentially important factor in determining whether a patient is at a high risk for diabetes (Adami and Bottai, 2020; Krentz and Gloyn, 2020).

**FIGURE 1:**
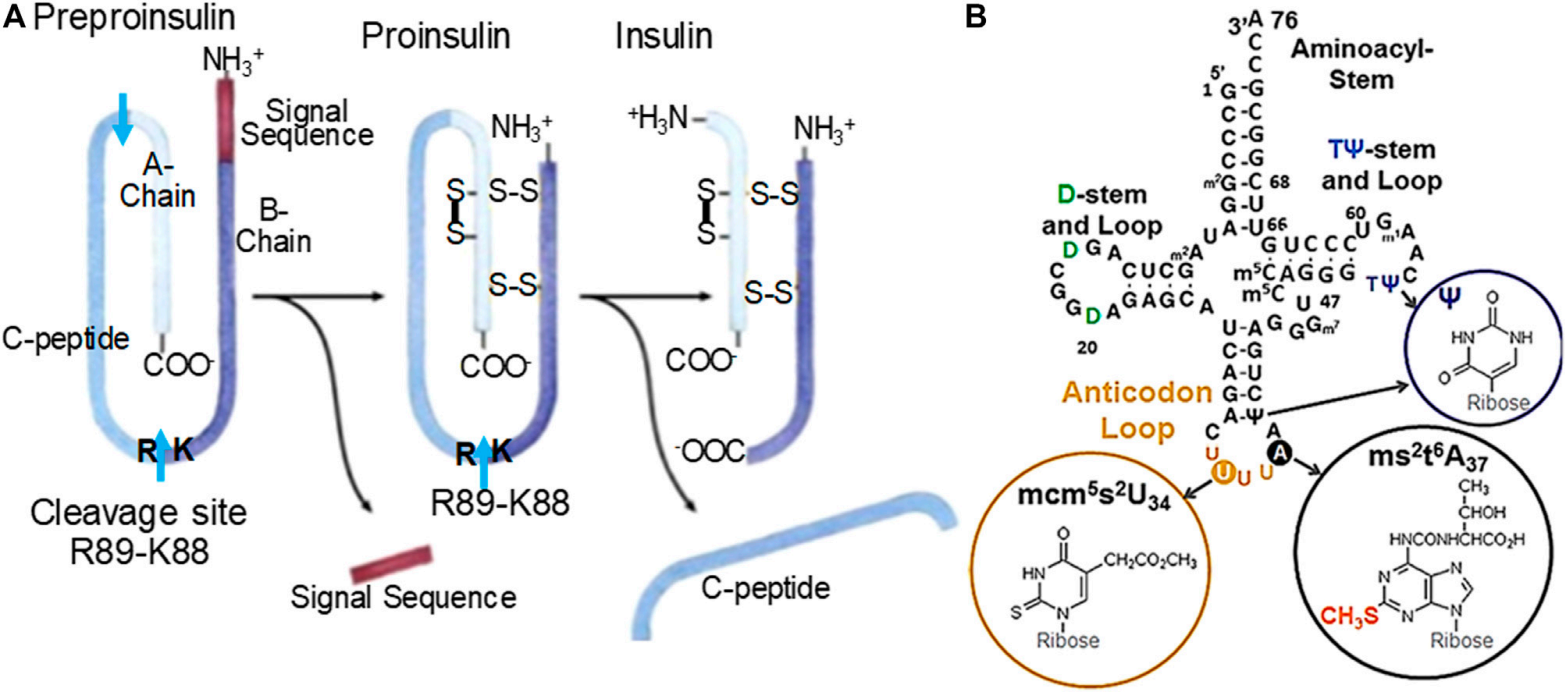
**(A)** Human insulin processing. Insulin mRNA is translated into one peptide that will undergo cleavage and linkages via disulfide bridges. One of two important cleavage sites is K88-R89. A preproinsulin mRNA necessitates tRNA^Lys3^ (B.) with its fully modified anticodon loop nucleosides to accurately read the Lys AAG codon. The resulting peptide is composed of a signal sequence, A-chain, B-chain and C-peptide, the A- and B-chain associate into mature insulin. **(B)** Human tRNA^Lys3^. tRNA^Lys3^ decodes AAA and AAG in mRNA on the ribosome. The ms^2^-modification of t^6^A_37_ results in ms^2^t^6^A_37_ and is the product of Cdkal1 enzymatic activity. The modification ms^2^t^6^A_37_ facilitates accurate and efficient decoding of the lysine codons. Domains are labeled. Anticodon loop modified nucleosides positions and structures are circled. The ms^2^-modification made by Cdkal1 is found with ms^2^t^6^A_37_. The anticodon 5′-adjacent invariant U and 3′-modified adenosine are noted.

Cdkal1 protein is a post-transcriptional tRNA modification enzyme, a methylthiotransferase which adds a methylthio (ms^2^-) moiety to the 2-position of an already modified adenosine-37, N^6^-threonylcarbamoyladenosine-37 (t^6^A_37_) in tRNA^Lys3^. The resulting ms^2^t^6^A_37_, 3′-adjacent to the anticodon (Figure 1), augments translational fidelity for the tRNA to bind the lysine codons AAA/G on the ribosome (Arragain et al., 2010; Agris et al., 2017). Only fully modified tRNA^Lys3^ is capable of accurately and efficiently decoding the AAA and AAG codons (Agris et al., 2017). Of the ∼five *cdkal* isoforms, only *cdkal1* is expressed in human islets and pancreatic cells in culture (Brambillasca et al., 2012). GWAS have found single nucleotide polymorphisms (SNPs) within intron-5 of the human *cdkal1* gene on human chromosome 6 that are considered a high risk for the disease (Diabetes Genetics Initiative of Broad Institute et al., 2007; Steinthorsdottir et al., 2007). The homozygous recessive mutation in the *cdkal1* gene has a 1.50 risk of T2DM, comparable to *brca1* and *2*. We found that the *cdkal1* gene is likewise critical to *Drosophila melanogaster*, the organism having insulin-like peptides. The gene *CG6550* catalyzes the methylthiolation of N^6^-threonylcarbamoyladenosine leading to the formation of ms^2^t^6^A_37_ in *Drosophila* tRNAs. The homozygous mutant *CG6550* (Df(2R)BSC44/P{EP}G7578), with the exception of rare escaper males, was lethal. Meta-analysis of genetic variations including homozygous recessive mutations in Cdkal1 shows that the occurrence of single nucleotide polymorphisms in the introns of Cdkal1 effect pre-mRNA processing events which impacts the production, processing, and availability of human insulin.

In β-cells, the protein Cdkal1 uniquely modifies just one of ∼45 human tRNA species, tRNA^Lys3^. tRNA^Lys3^ is one of the three tRNAs for the amino acid lysine. Cdkal1 is a member of the iron-sulfur cluster enzymes that use the substrate S-adenosylmethionine (Forouhar et al., 2013; Landgraf et al., 2016). Recently and understandably, iron-deficiency has been associated with T2DM through Cdkal1 under-performing for lack of iron (Santos et al., 2020). The AAG codon in human pre-proinsulin mRNA codes for both Lys53 and Lys88. Importantly, Lys88 is positioned adjacent to Arg89 and establishes the point at which a crucial protease cleavage separates the insulin A-chain from the C-peptide. Thus, in *cdkal1*-associated T2DM it is believed that a non-functioning or missing Cdkal1 protein in islet β-cells would result in tRNA^Lys3^ lacking the modification ms^2^t^6^A_37_. The tRNA^Lys3^ in turn would not be able to insert lysine at position 88 in response to the AAG codon. As a result, proinsulin would not be cleaved into insulin. In this study, we knocked down the *cdkal1* gene in cell culture to show the effect of Cdkal1 silencing in pre-mRNA processing and production of mature insulin. The result was not only the *cdkal1* mRNA and Cdkal1 protein were decreased, but mature insulin was also reduced. The tRNA modification ms^2^t^6^A_37_ was reduced relative to its precursor, t^6^A_37_ in NIT-1 cells. We are first to demonstrate why tRNA^Lys3^ lacking the ms^2^-modification of ms^2^t^6^A_37_ is unable to decode the lysine wobble codon AAG.

## Materials and Methods

### Cell Line

NIT-1 is a β-cell line established from a transgenic mouse with the SV40 large T-antigen. It is grown in Hams F12K medium (F12K with L-Glutamine 90%; heat-inactivated, dialyzed fetal bovine serum 10%, FBS, Sigma; and 1 X penicillin-streptomycin, Invitrogen). Theophylline (10 mM with 5.5 mM glucose), glucose or KCl were used to stimulate insulin production. To knockdown the gene, we transfected an experimental esiRNA (endonuclease-prepared siRNA, Sigma) and a control GFP esiRNA with Lipofectamine 2000 and a Lentiviral shRNA (Sigma).

### Stimulation of Insulin Production

NIT-1 has a glucose stimulated insulin response (GSIR) generated with a high glucose concentration (25 mM) when preceded by overnight incubation in low glucose, serum-free medium (SF-DMEM). Insulin production was assayed by ELISA (mouse proinsulin and insulin antibody, ABClonal).

### Transfections

Plated cells were transfected with a control GFP esiRNA or cdkal1 esiRNA (30, 50 or 70 nM using Lipofectamine, LF, RNAiMax reagent, 0.5 or 1 μL, Invitrogen) in antibiotic-free medium (6 h). Cells were then incubated in fresh medium (48 h), serum and glucose starved overnight in SF-DMEM, and induced for insulin (90 min) or left unstimulated (SF-DMEM with 600 KIU/ml aprotinin).

### Modified Nucleoside Analysis

Small RNAs (<200 nucleotides) were isolated (Ambion mirVana miRNA Isolation Kit) and the RNA was dialyzed extensively against phosphate buffer (10 mM NaH_2_PO_4_, pH 6.8) and then against water (18 mΩ). The RNA was hydrolyzed to nucleosides enzymatically rather than chemical digestion. The 2-step process cleaves first the phosphodiester bond with nuclease P1 resulting in nucleoside-5′-monophosphates followed by bacterial alkaline phosphatase (BAP) to cleave the 5′-phosphate from the nucleosides resulting in individual nucleosides and phosphoric acid. The modified nucleoside analysis was conducted by UHPLC-MS/MS (triple quadrupole MS (Waters MS) (Basanta-Sanchez et al., 2016).

### RT-qPCR of Insulin and *cdkal1* mRNA

We used RT-qPCR to assess the level of expression of mature insulin (mouse insulin I and II) or a pre-insulin or precursor containing intron 2 in normal and in esiRNA transfected NIT-1 cells, induced and not induced for insulin production. Large sequence RNA was isolated from normal and transfected knockdown NIT-1 cells (Qiagen RNEasy Plus kit) and rRNA was removed (Qiagen RNEasy MinElute Kit). A 2-step RT-PCR was conducted to determine the presence of *cdkal1*, precursor insulin, and mature insulin mRNAs (BioRad 2step c-DNA Synthesis kit) (Iype et al., 2005). The expression and amount of *cdkal1* mRNA was determined by quantitative real-time PCR (RT-qPCR) analysis. Threshold Cq values were normalized to actin levels. Relative expression was calculated using the 2^ΔΔCq^ method (Livak and Schmittgen, 2001).

### Molecular Dynamics Simulations

The crystal structure of the mammalian ribosome was obtained from the Protein Data Bank (PDB ID: 5LZS) (Shao et al., 2016). An intact stable fragment of structure was used for simulations, which included the mRNA, the anticodon stem loop (ASL) of the A-site tRNA, ribosomal RNA and ribosomal proteins within 25 Å of the codon and anticodon minihelix at the A-site. The ASL and the mRNA codon were modified to match the human tRNA^Lys3^ ASL sequence and the lysine codon respectively using MOE (Chemical Computing Group, 2019). Six different constructs of the ASL:codon pair were modeled with codons AAA and AAG, each paired with the ASL containing the unmodified nucleoside A_37_, N^6^-threonylcarbonyladenosine (t^6^A_37_) and hypermodified 2-methylthio N^6^-threonylcarbonyl adenosine (ms^2^t^6^A_37_).

In order to simulate the modified tRNA, AMBER (Cornell et al., 1995) type force-field parameters were developed for the atoms of the modified nucleosides—pseudouridine Ψ, mcm^5^s^2^U, t^6^A and ms^2^t^6^A. The geometry of the modified nucleosides was optimized using Hatree–Fock level theory and 6-31G* basis-sets in Webmo (Schmidt and Polik, 2020). For obtaining the partial charges on the atoms, the online RESP charge-fitting server REDS was used (Cornell et al., 1993; Dupradeau et al., 2010). AMBER-99 force field parameters and AMBER-99 parameters with the Chen–Garcia correction were used for bonded and Lennard–Jones (LJ) interactions, respectively (Cornell et al., 1995; Chen and García 2013).

Molecular dynamics (MD) simulations were performed using Gromacs-2016.4 and Gromacs-2019.6 packages (Abraham et al., 2015). The MD simulations incorporated a leap-frog algorithm with a 2-fs timestep to integrate the equations of motion. The system was maintained at 300 K, using the velocity rescaling thermostat (Bussi et al., 2007). The pressure was maintained at 1 atm using the Berendsen barostat for equilibration (Berendsen et al., 1998; Parrinello and Rahman 1998). Long-range electrostatic interactions were calculated using particle mesh Ewald (PME) algorithm with a real space cut-off of 1.0 nm (Darden et al., 1998). LJ interactions were truncated at 1.0 nm. The TIP3P model was used to represent the water molecules, and the LINCS algorithm was used to constrain the motion of hydrogen atoms bonded to heavy atoms (Bekker et al., 1997). The system was subjected to energy minimization to prevent any overlap of atoms, followed by 0.5 ns of equilibration and a 25-ns production run. During simulations, the ribosomal RNA, proteins and the mRNA (except the codon) were held in place using position restraints on the heavy atoms of the RNA and protein backbone with a force constant of 1,000 N/nm in each spatial dimension for the simulation. Coordinates of the ribosomal fragment (rRNA, tRNA, and mRNA) were stored every 1 ps for further analysis. The simulations were visualized using Visual Molecular Dynamics software and analyzed using tools from Gromacs (Humphrey et al., 1996; Abraham et al., 2015).

## Results

**FIGURE 2:**
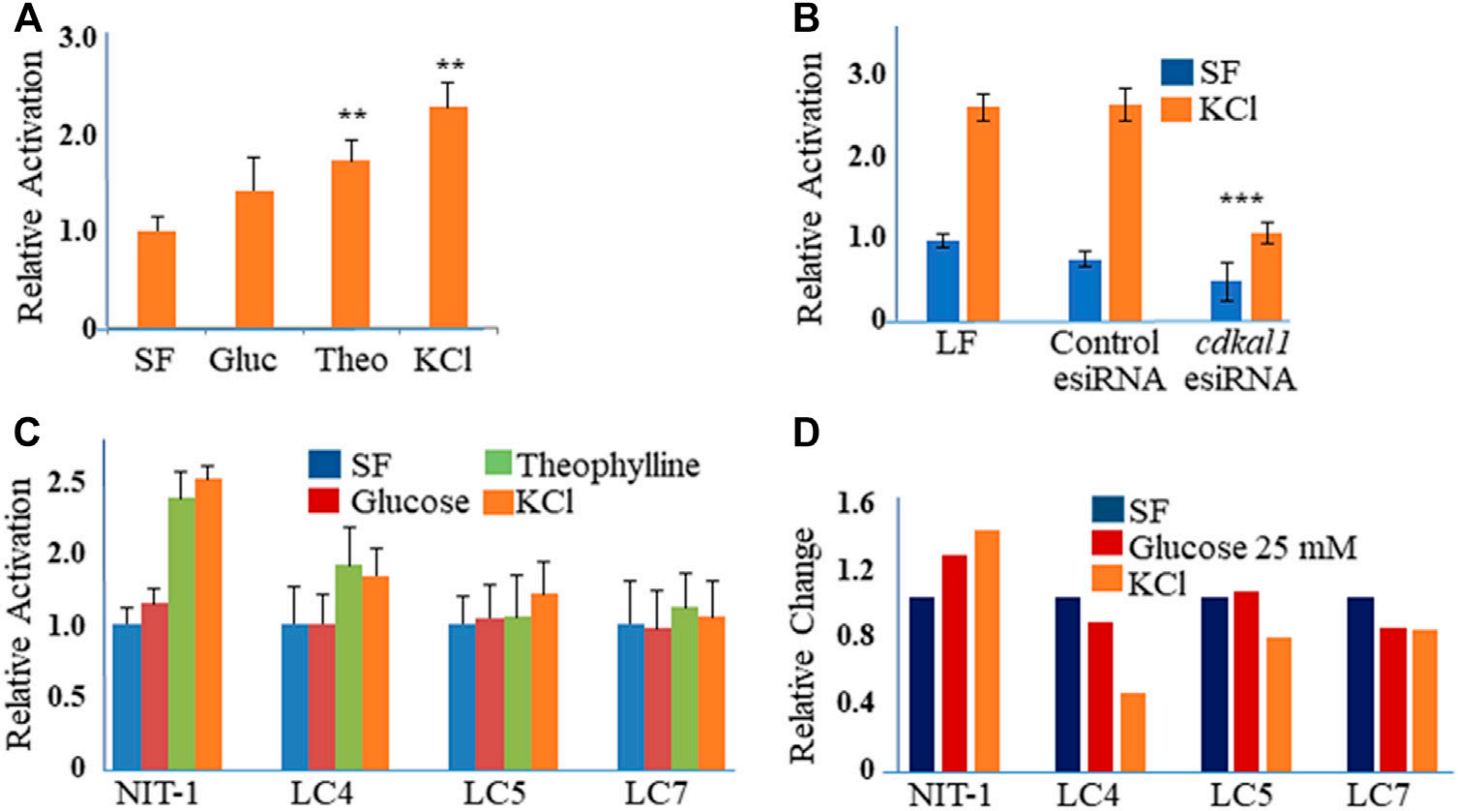
**(A)** Relative activation of insulin produced in stimulated NIT-1 cells. NIT-1 cells plated in 24 wells for 48 h were serum starved overnight and stimulated for 90 min with control (SF), Glucose (25 mM), Theophylline (10 mM in Glu 5.5), and KCl (40 mM) in SF medium with Aprotinin. **(A)** Insulin secretion in cell supernatants was measured by ELISA using a mouse proinsulin and insulin antibody and concentration calculated using a human insulin protein standard curve. Insulin levels were normalized to control cells and expressed as fold-change over control (Relative Activation). Results are an average three biological experiments. **Indicates *p* value < 0.01 using a Students t-test. **(B)** Insulin levels in supernatants from NIT-1 knockdown cells. Secreted insulin levels from esiRNA-transfected (50 nM with LF 1 µL) NIT-1 cells were measured by ELISA following stimulation with KCl or Serum-free medium. Data is expressed as Relative Activation compared to unstimulated (SF) Lipofectamine RNAiMAx (LF)—only transfected control cells. ***Indicates *p* value < 0.001 using a Students t-test. **(C)** Secreted insulin levels from Lentiviral shRNA knockdown clones of NIT-1 cells (LC4, LC5 and LC7) were measured by ELISA following stimulation. Data is expressed as Relative Activation compared to non-stimulated control cells of each cell type. Experiments were controlled for the amounts of protein. **(D)** Insulin protein levels in esiRNA knockdown cell lysates. Protein levels were normalized to GAPDH and expressed as Relative Densitometric Units (RDU).

The NIT-1 cell line (ATCC® CRL-2055™) is inducible for insulin production with high glucose concentrations, theophylline and KCl (Figure 2A). The cells were stimulated to produce insulin with Theophylline (10 mM with 5.5 mM glucose) or KCl (40 mM) in SF media (Figure 2A). In order to determine the effect of a reduced Cdkal1 protein production, we knocked down *cdkal1* gene expression with two distinct methods. The *cdkal1* gene was knocked down by transfection of an esiRNA (endonuclease-prepared siRNA, 30, 50 or 70 nM using Lipofectamine, LF; Sigma) and by a Lentiviral shRNA (Sigma)*.* Cells were also transfected with a control GFP esiRNA in antibiotic-free medium (6 h). Cells were glucose starved overnight in SF-DMEM, and induced for insulin (90 min) or left unstimulated (SF-DMEM with 600 KIU/ml aprotinin). Cells transfected with a control GFP esiRNA or Lipofectamine (LF RNAiMax reagent) alone responded to stimulation similar to cells that had not been transfected. Supernatants of *cdkal1* esiRNA (50 nM) knockdown cells assayed for insulin by ELISA had as low as 20% of the mature insulin production compared to a control esiRNA and LF-transfected cultures (Figure 2B). The cells were lysed in RIPA buffer with protease inhibitors and the lysates were processed for Western blots that showed decreased Cdkal1 protein as well as decreased insulin production (Supplemental Figure S1).

Cells were also infected with four different Lentiviral shRNA constructs against the *cdkal1* gene in antibiotic-free medium and selected with puromycin (3.5 µg). Clones, LC4, LC5, and LC7 that survived puromycin selection were expanded and DNA isolated and sequenced. Insulin induced cells were analyzed with the GSIR assay, ELISA and Western blot. Compared to normal NIT-1 cells, the secreted insulin levels were 50% or lower with stimulation by Theophylline and KCl (Figure 2C). When cell lysates were assayed for insulin, LC4 stimulated by KCl showed that the knockdown of *cdkal1* by the shRNA construct inhibited insulin production by >60% (Figure 2D). Analysis of stimulated NIT-1 cell lysates by Western blotting showed no significant changes in the levels of Cdkal1 protein in normal NIT-1 cells although knockdown cells had lower Cdkal1 protein levels (Supplemental Figure S1). The amount of GAPDH control protein was not affected in the Cdkal1 deficient cells.

**FIGURE 3:**
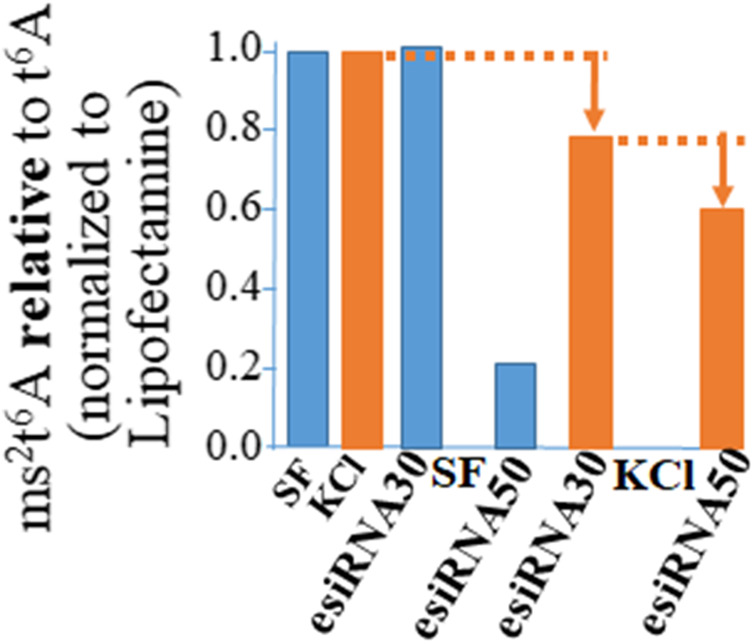
The ms^2^t^6^A modification is diminished in tRNA from *cdkal1* esiRNA knockdown cells. The modified nucleosides in small RNA purified from esiRNA30 and esiRNA50 knockdown cells and control LF cells were hydrolyzed to nucleosides and analyzed by UHPLC-MS/MS. The results are presented as the amount of ms^2^t^6^A relative to ms^2^t^6^A plus t^6^A (ms^2^t^6^A/ms^2^t^6^A + t^6^A) normalized to LF controls. Initially the concentrations of ms^2^t^6^A and t^6^A were determined relative to the major nucleoside A. The results are the average of three biological assays conducted by two different institutions. LF—control cells transfected with Lipofectamine only; esiRNA30 and 50—Cdkal1 knockdown cells transfected with 30 and 50 nM esiRNA. SF—control cells serum-free; KCl—4 mM KCl stimulated.

The function of Cdkal1 protein’s modification of tRNA is accurate and efficient translation of AAG/AAA codons. Insulin mRNA requires the Cdkal1 modification of tRNA^Lys3^ from t^6^A_37_ to ms^2^t^6^A_37_ for insertion of lysine at position 88. A significant decrease was observed in ms^2^t^6^A relative to t^6^A in RNA (<200 nts) isolated from cells that had been knocked down (Figure 3). We isolated RNA from esiRNA knockdown cells, stimulated and unstimulated for insulin production and control Lipofectamine only (LF) cells. The RNA was fractionated so that we could analyze small RNAs less than 200 nucleotides such as tRNAs without rRNA present (Ambion mirVana miRNA Isolation Kit). The RNA was hydrolyzed to nucleosides and the modified nucleoside analysis conducted by UHPLC-MS/MS (triple quadrupole MS (Waters MS). The ms^2^t^6^A modification decreased in tRNAs from esiRNA30 and esiRNA50 knockdown cells. As expected, the decrease was most dramatic in tRNA from esiRNA50 cells grown in SF medium, and less so when the cells were stimulated by KCl. The decrease of ms^2^t^6^A in tRNA is consistent with and probably the cause of a decrease in secreted insulin and increase in precursor insulin mRNA.

**FIGURE 4:**
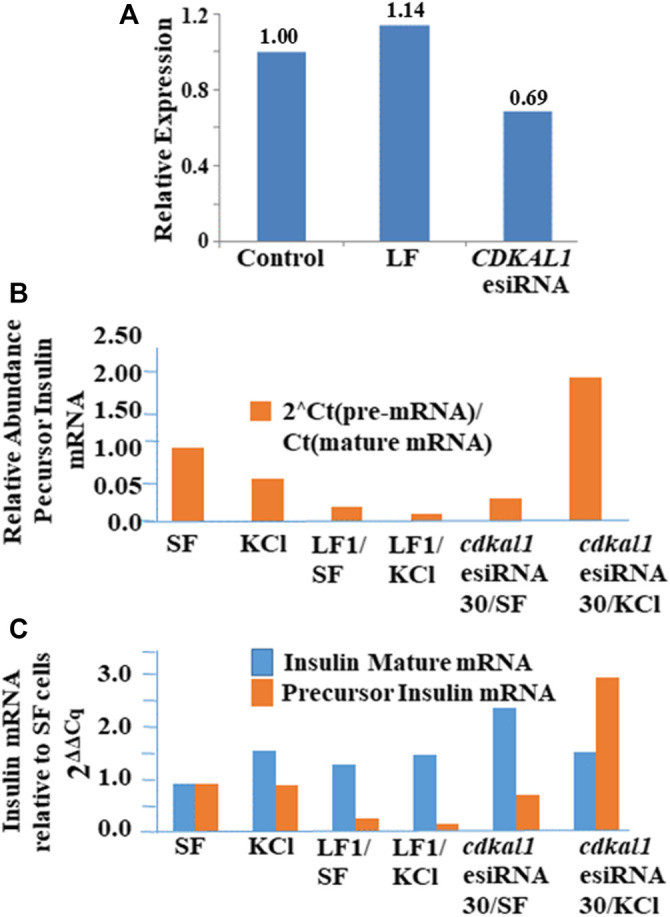
Insulin, precursor insulin and Cdkal1 mRNA levels in *cdkal1* esiRNA knockdown cells. **(A)** Relative expression of insulin mature mRNA relative to pre-mRNA is increased with stimulation in normal NIT-1 cells. **(B)** Mature insulin mRNA is significantly reduced in KCl stimulated knockdown cells. mRNA levels were normalized to actin mRNA and expressed as fold-change to Control normal NIT-1 cells. LF cells—transfected with Lipofectamine only, esiRNA30—transfected with esiRNA 30 nM. In *cdkal1*-esiRNA treated knockdown cells, the mRNA levels were reduced by ∼30%. Threshold Cq values were normalized to actin levels. Relative expression was calculated using the 2^ΔΔCq^ method. **(C)** The insulin mature and precursor mRNA levels were assessed with RT-qPCR. RNA isolated from normal or knockdown NIT-1 cells following stimulation for insulin production. The 2-step RT-qPCR method was used to determine the expression levels of insulin mRNA. Threshold Cq values were normalized to actin levels. Expression relative to SF mRNA levels was calculated using the 2^ΔΔCq^ method. The abundance of precursor insulin mRNA relative to mature mRNA increased in KCl-stimulated NIT-1 cells when *cdkal1* was knocked down with esiRNA. To measure the transcription of the insulin gene, we used RT-qPCR to assess the expression of total or mature insulin (mouse insulin I and II) or a pre-insulin or precursor containing intron-2 in normal or esiRNA transfected NIT-1 cells. (BioRad 2-step RT-PCR Kit). Forward and reverse primers used to amplify mature insulin mRNA (5′-TGGCTTCTTCTACACACCCAAG-3′ and 5′-ACAATGCCACGCTTCTGCC-3′), insulin pre-mRNA (5′-GGGGAGCGTGGCTTCTTCTA-3′ and 5′-GGGGCGATTCAGTGGCA-3′), or *actin* (5′-AGGTCATCACTATTGGCAACGA-3′ and 5′-CACTTCATGATGGAATTGAATGTAGTT-3′). PCR cycles were at 95°C for 15 s, 64°C for 1 min for 50 cycles. Melt curve analysis confirmed the homogeneity of products from each reaction. Relative abundance of pre-insulin was calculated using the formula 2[*CT*(mat mRNA)-*CT*(pre-mRNA)].

Large sequence RNA was isolated from normal and transfected knockdown NIT-1 cells (Qiagen RNEasy Plus kit) and rRNA was removed (Qiagen RNEasy MinElute Kit). Quantitative real-time PCR was conducted to determine the presence of *cdkal1*, precursor insulin, and mature insulin mRNAs. Threshold Cq values were normalized to actin levels. Relative expression was calculated using the 2^ΔΔCq^ method. The expression of *cdkal1* mRNA levels increased by ∼60% in normal NIT-1 cells following stimulation with KCl (Figure 4A). However, in stimulated *cdkal1*-esiRNA treated knockdown cells, the mRNA levels were decreased by ∼30% (Figure 4B). To determine the regulation of insulin transcription, we applied a unique strategy using two sets of primers that amplified total mouse insulin I and II mRNA (fully processed mature insulin mRNA) and a precursor mRNA species containing intron 2 (Figure 4C). The levels of mature mRNA and precursor mRNA insulin levels were quantitated and expressed as the real time threshold cycle (C_T_) values, in untreated (SF) and KCl stimulated NIT-1 normal or knockdown cells. Although no differences were seen in mature insulin mRNA levels, significant changes in precursor insulin mRNA levels were detected with stimulation to produce insulin (Figure 4C). When the abundance of precursor insulin mRNA relative to mature insulin mRNA in control cells is normalized to 1.00, in stimulated *cdkal1* knockdown cells the ratio is 1.95 relative to mature insulin mRNA. In β-cells when the *cdkal1* gene is non-functional or missing and the tRNA^Lys3^ modification ms^2^t^6^A has decreased, *cdkal1* mRNA has decreased 30% also. However, the insulin precursor mRNA is significantly increased. Thus, *cdkal1* knockdown cells stimulated to produce insulin are yet secreting less mature insulin. Lysates from these cells exhibited significantly decreased insulin and proinsulin (Figure 2D).

We asked why should the modification ms^2^-play such an important role in tRNA^Lys3^ in translating the lysine wobble codon AAG in insulin mRNA? When the anticodon U_34_U_35_U_36_ with the adjacent ms^2^t^6^A_37_ binds the wobble codon G3A2A1, the ms^2^t^6^A_37_ is three nucleosides distant from the U_34_:G3 pair. There are three posttranscriptional modifications in the anticodon stem and loop (ASL) of tRNA^Lys3^, 5-methoxycarbonylmethyl-2-thiouridine at wobble position 34 (mcm^5^s^2^U_34_), 2-methylthio-*N* ^*6*^-threonylcarbamoyladenosine at position 37 (ms^2^t^6^A_37_) adjacent to the anticodon and pseudouridine (Ψ_39_) at position 39 in the stem. The fully modified ms^2^t^6^A_37_ and mcm^5^s^2^U_34_ are required to achieve wild-type binding activity of human tRNA^Lys3^ to AAA and the wobble codon AAG (Yarian et al., 2000). NMR structure determination and molecular dynamics simulations (MDS) of the ASL demonstrated that the ms^2^t^6^-modification of A_37_ supports the anticodon nucleoside stack 5′ to 3′ and reduces solvent accessibility of U_36_ (Stuart et al., 2000; McCrate et al., 2006).

**FIGURE 5:**
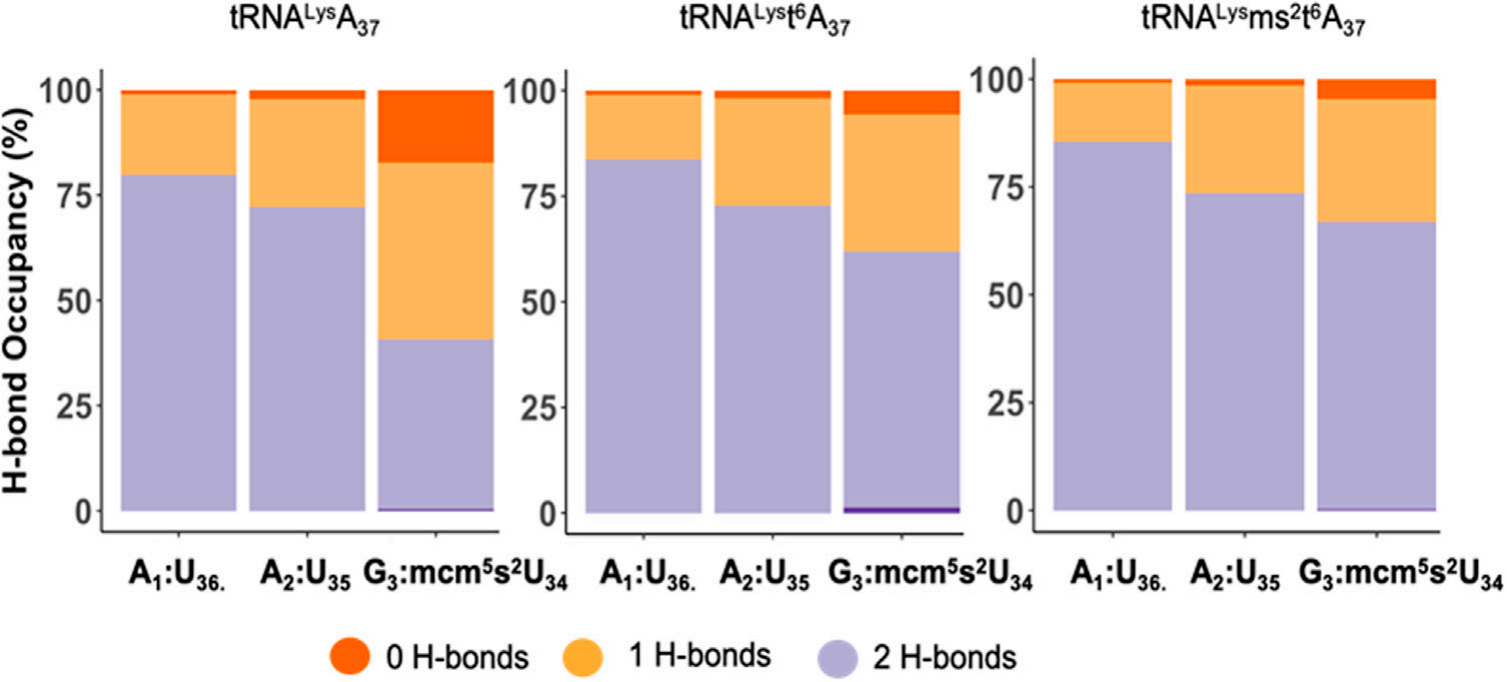
The hydrogen bond occupancy for the tRNA^Lys3^ anticodon-codon base pairs. The hydrogen bond (H-bond) occupancy for the tRNA^Lys3^ anticodon-codon base pairs for the mcm^5^U_34_U_35_U_36_ anticodon interacting with the A_1_A_2_G_3_ wobble codon for the three cases of tRNA^Lys3^-A_37,_ tRNA^Lys3^-t^6^A_37_ and tRNA^Lys3^-ms^2^t^6^A_37_. The chart presents the H-bond occupancy for the percentage of the simulation time the base-pairs, A1:U_36,_ A2:U_35_, G3:mcm^5^s^2^U_34_ interact and establish 0, 1, or 2 H-bonds.

To explore the role of the tRNA^Lys3^ modifications at A_37_ for recognition and decoding, we performed molecular simulations of the anticodon stem-loop of the tRNA (ASL) bound to the mRNA AAG at the A site of the eukaryotic ribosome. We compared three simulations with the ASL-mcm^5^s^2^U_34_ with A_37_, t^6^A_37_, and ms^2^t^6^A_37_ each bound to the wobble codon AAG on the ribosome. First, we considered the effect of the modifications on the codon-anticodon interaction. We compared the hydrogen bonding between the codon and anticodon nucleosides (A1:U_36_, A2:U_35_ and G3:mcm^5^s^2^U_34_), in the three systems (Figure 5). Interestingly, we observe that the hydrogen bonding is stronger for all three positions of the codon-anticodon base pairs by the addition of the t^6^-modification to A_37_ and is further enhanced by the addition of the ms^2^- to t^6^A_37_. Remarkably, we find that this enhancement is most pronounced when the mcm^5^s^2^U_34_:G_3_ base-pair is considered, which is the farthest from the A_37_.

**FIGURE 6:**
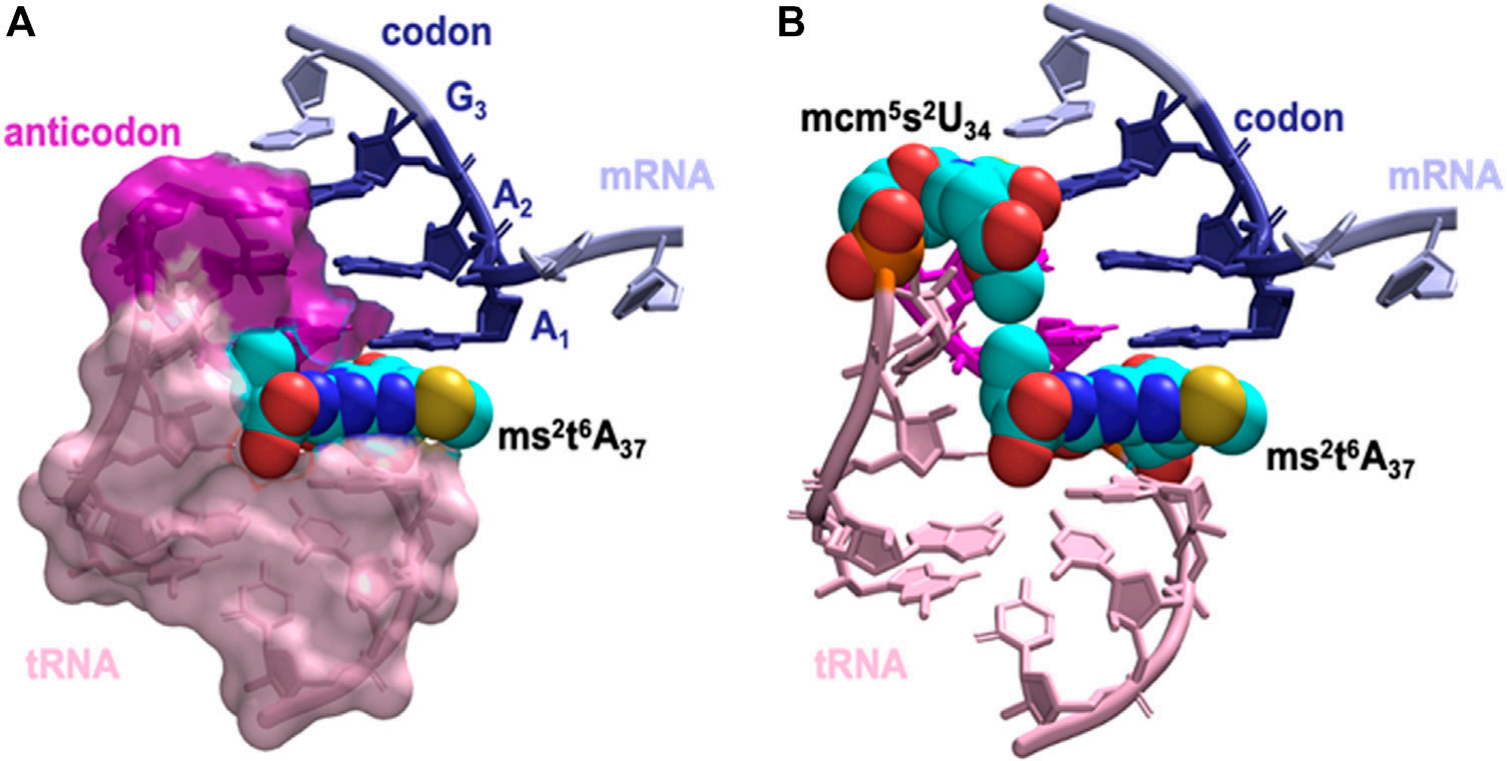
The dominant orientation of the hypermodified ms^2^t^6^A_37_ shown in space filling model interacting with the anticodon stem loop cavity. **(A)** The terminal carboxyl group of the threonylcarbamoyl-group faces away from the ASL cavity, while the aliphatic carbons and the terminal methyl group fill up the cavity. Stacking between A1, A_37_ and A_38_ is enhanced by the addition of the thiomethyl-group to t^6^A_37_, ms^2^t^6^A_37_. **(B)** The interaction between hypermodified ms^2^t^6^A_37_ and mcm^5^s^2^U_34_ shown in space filling model. Hydrophobic contacts between the two modified nucleotides assists mcm^5^s^2^U_34_ in maintaining the anticodon:codon interaction at the wobble position.

Next, we asked how does the modification at A_37_ lead to significant strengthening of codon anticodon base-pairing? The dominant locations of the threonylcarbamoyl-group in both t^6^A_37_ & ms^2^t^6^A_37_ systems has the hydrophilic moieties of the modification (carboxyl and hydroxyl groups) either pointing away from the ASL cavity and remaining well hydrated or are involved in a cross-loop interaction with the backbone (2′ hydroxyl group) of C_32_ (Figure 6A). The rest of the modification fits inside the ASL cavity through hydrophobic and hydrogen bonding interactions, thereby offering stability to the neighboring codon-anticodon base-pairs. Furthermore, we observed transient interaction between the terminal methyl groups of ms^2^t^6^A_37_ and mcm^5^s^2^U_34_ (Figure 6B), suggesting that the enhancement in stability due to the modification at A_37_ extends to the codon-anticodon base pair farthest from A_37_. Most interestingly, we also found that the t^6^-group interaction with the ASL cavity is more stable with the addition of the ms^2^-group (see Movie, t^6^A_37_ in pink and ms^2^t^6^A_37_ in yellow). The ms^2^-group boosts the stacking interaction between A_37_ and the A1 codon, as a result of which the threonylcarbamoyl-group is held steady in the ASL cavity.

Overall, our molecular simulations reveal a cascading mechanism for ms^2^t^6^A_37_, in which hydrogen bonding energy and the hydrophobic interactions of base-stacking by the methylthio-group stabilizes the threonylcarbamoyl-group in the ASL cavity. This in turn facilitates the hydrophobic interaction of the threonylcarbamoyl-group with the methylcarboxymethyl- (mcm^5^-) group on U_34_ three nucleosides away, stabilizing the codon-anticodon base-pairing at the wobble position for wobble codon AAG recognition.

## Discussion

Homozygous recessive mutations in the human *cdkal1* gene such as SNPs in intron-5 are a significant risk for T2DM. Several population studies showed the significant role of SNPs in the development of T2DM in different races, but the overall available data is not sufficient to reveal the biochemical role of particular SNPs to their concerned roles towards the development of diabetes. A *cdkal1*-deficient mouse presents properties characteristic of human T2DM (Wei et al., 2011). In this study, we investigated the consequences of diminished function of Cdkal1 in pancreatic islets cell NIT-1 cell lines and uncovered a biochemical connection of the tRNA modification enzyme to insulin translation and processing. SNPs in intron-5 of *cdkal1* could cause alternative mRNA splicing (Zhou et al., 2014) and a reduction of Cdkal1 protein synthesis. Here, diminished function of Cdkal1 by knockdown of the *cdkal1* gene in mouse NIT-1 cells resulted in not only the reduction of tRNA^Lys3^ modification, but also a decrease in insulin mRNA and mature insulin. Using MDS we were able to determine that the tRNA^Lys3^ lacked hydrogen bonds and stacking to the wobble codon AAG when missing the ms^2^-modification of t^6^A_37_. Generation of *cdkal1* knockout mouse (Shao et al., 2016) and availability of knockout cell lines increases the possible ways to study the quite challenging SNPs in genetic level to reveal its functional roles in mature insulin production and availability.

## Data Availability

The raw data supporting the conclusions of this article will be made available by the authors, without undue reservation.

## Supplementary Material

The Supplementary Material for this article can be found online at: https://www.frontiersin.org/articles/10.3389/fmolb.2020.584228/full#supplementary-material.**Supplementary Figure S1. Insulin B protein and Cdkal1 protein in NIT‐1 knockdown cell lysates**. Western blots were employed to identify levels of insulin, Cdkal1 and control GAPDH proteins in Control esiRNA (70 nM) and *cdkal1* esiRNA knockdown cells unstimulated (Serum Free, SF) and stimulated (KCl) to produce insulin. **A**. Cdkal1 levels in control LF and GFP esiRNA cells stimulated and not stimulated to produce insulin and in *cdkal1* esiRNA (70 nM) knockdown cells. **B**. Insulin B protein levels in control LF and GFP esiRNA-treated cells stimulated and not stimulated to produce insulin and in *cdkal1* esiRNA (70 nM) knockdown cells. C. Mouse antibodies were used in Western blots against Insulin B and Cdkal1 or GAPDH. Insulin production was induced by low and high glucose, Tolbutamide, Theophyllin and KCl. Protein levels in cell lysates in Western blots were quantitated with Image J software and normalized to GAPDH levels and expressed as fold-change from control unstimulated (SF) cell lysates. LF – Lipofectamine (1ul); Control *GFP*‐esiRNA 70 nM; *cdkal1*‐esiRNA 70 nM. Protein (250 μg) was loaded on SDS‐PAGE gels and immune blotted using mouse antibodies against Insulin B (Clone C‐12, Santa Cruz), Cdkal1 (clone E9, Santa Cruz), or GAPDH (Clone MA5‐GA1R, Thermo Fisher). GAPDH was used as a loading control.
