# Supplementary figures and images for: Silencing of the tRNA Modification Enzyme Cdkal1 Effects Functional Insulin Synthesis in NIT-1 Cells: tRNALys3 Lacking ms2- (ms2t6A37) is Unable to Establish Sufficient Anticodon:Codon Interactions to Decode the Wobble Codon AAG
Source: Front Mol Biosci. 2021 Feb 9;7:584228. doi: 10.3389/fmolb.2020.584228 (PMC7900539; doi:10.3389/fmolb.2020.584228)

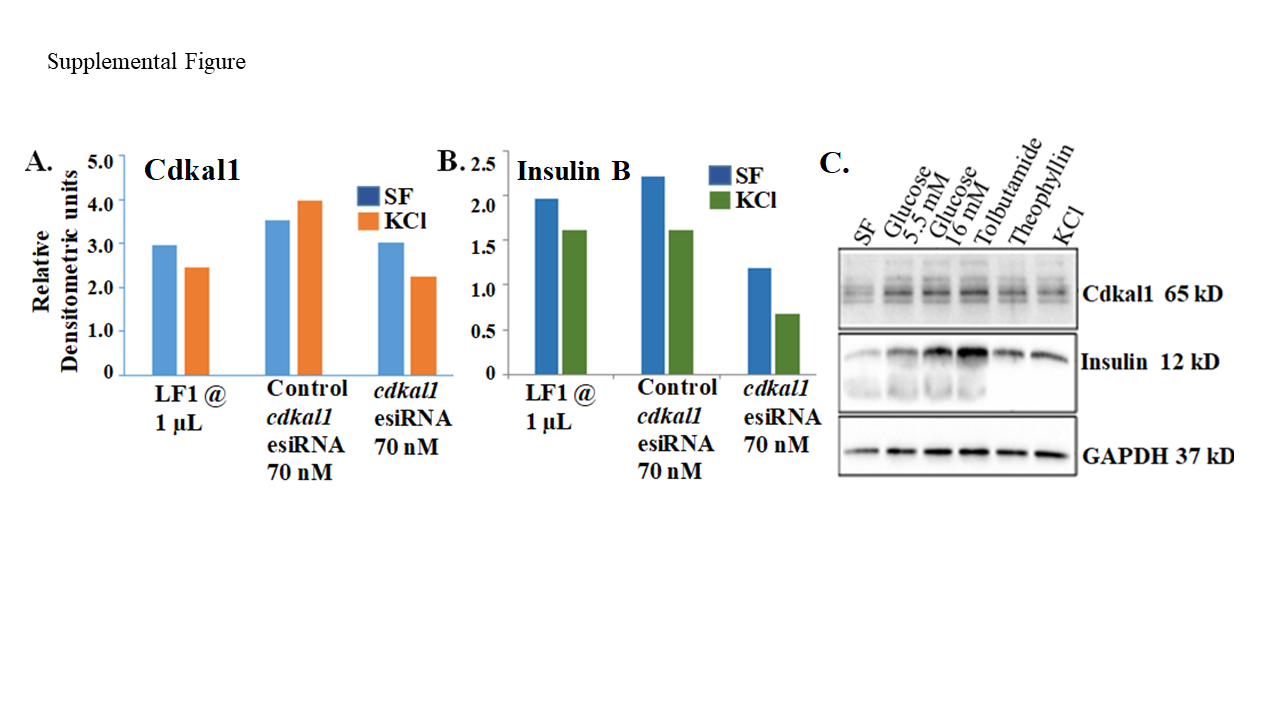

Supplement: Supplementary file 2 [file image1.tif]
